# Diabetic kidney disease: emerging mechanistic and nutritional perspectives from the Mediterranean diet–mitochondria axis. A narrative review

**DOI:** 10.1016/j.jnha.2026.100987

**Published:** 2026-09-15

**Authors:** Giovanna Mercurio, Antonia Giacco, Nicla Scopigno, Michela Vigliotti, Giuseppe Petito, Maria Moreno, Federica Cioffi, Elena Silvestri

**Affiliations:** a Department of Science and Technology, University of Sannio, Benevento, Italy; b Dipartimento di Scienze e Tecnologie Ambientali, Biologiche e Farmaceutiche, Università Degli Studi Della Campania “Luigi Vanvitelli”, Caserta, Italy

**Keywords:** Cell type–specific mitochondrial dysfunction, Mitochondrial quality control, Nutritional nephroprotection, Dietary polyphenols

## Abstract

**Objectives:**

To examine mitochondrial dysfunction as a key driver of diabetic kidney disease (DKD) and to evaluate the Mediterranean diet (MD) as a potential nutritional modulator of renal mitochondrial health.

**Methods:**

Clinical, epidemiological, and preclinical studies addressing mitochondrial alterations in podocytes and proximal tubular cells were critically reviewed, with particular attention to MD adherence and MD-derived bioactive compounds.

**Results:**

Evidence indicates that early, cell-specific mitochondrial dysfunction contributes to DKD progression through impaired bioenergetics, oxidative stress, altered mitochondrial dynamics, defective mitophagy, and reduced biogenesis. Clinical and epidemiological studies consistently associate higher MD adherence with preserved renal function and lower DKD risk, while experimental studies suggest that MD-derived bioactives modulate mitochondrial quality control pathways in renal cells.

**Conclusion:**

The MD–mitochondria axis represents a biologically plausible framework linking nutrition and mitochondrial homeostasis in DKD. Mediterranean diet adherence may therefore contribute to renal resilience and complement current strategies aimed at preventing DKD progression.

## Highlights

• Cell-specific mitochondrial dysfunction is an early driver of DKD in podocytes and tubules
• Mediterranean diet adherence associates with preserved renal function in DKD
• Mediterranean diet -derived bioactives target mitochondrial quality control pathways in podocytes and tubular cells
• Mediterranean diet emerges as a mitochondria-centered nutritional framework for DKD prevention

## What is known about this research topic?

• Mitochondrial dysfunction is an early and central driver of DKD, affecting both podocytes and proximal tubular cells.
• Adherence to the Mediterranean diet is associated with reduced DKD risk and slower decline in renal function.

## What this study adds and its future implications

• • This review integrates clinical and mechanistic evidence to link Mediterranean diet adherence with cell-specific mitochondrial pathways in DKD.
• • MD-derived bioactives converge on mitochondrial quality control processes, including redox balance, dynamics, mitophagy, and biogenesis.
• • Targeting the MD–mitochondria axis may inform future nutritional and preventive strategies complementing pharmacological DKD therapies.

## 1 Introduction

Diabetic kidney disease (DKD), formerly referred to as diabetic nephropathy (DN), represents one of the most serious complications of diabetes and accounts for a substantial proportion of chronic kidney disease (CKD) worldwide. It affects approximately 30% of individuals with type 1 diabetes and nearly 40% of those with type 2 diabetes (T2D), and remains a leading cause of kidney failure requiring dialysis or transplantation [1]. Beyond renal impairment, DKD markedly increases the risk of cardiovascular events and premature mortality [2].

The onset and progression of DKD arise from sustained disturbances in glucose metabolism that activate a complex network of metabolic, hemodynamic, inflammatory, and fibrotic pathways. Hyperglycemia-driven oxidative stress, chronic inflammation, apoptosis, and dysregulated autophagy contribute to tubulointerstitial inflammation and fibrosis, glomerular hypertrophy, glomerulosclerosis, and ultimately loss of renal function [3].

Although DKD was traditionally viewed as a primarily glomerular disease, emerging evidence now supports a major role for tubular pathology—particularly within the proximal tubule, a highly mitochondria-depending renal compartment—as an early and potentially initiating driver of renal decline [4]. This “tubular-centered” paradigm highlights mitochondrial dysfunction, oxidative stress, and impaired cellular quality control as key contributors to early DKD pathophysiology.

Overall, urinary production is a highly energy-demanding process that relies almost entirely on ATP generated through mitochondrial oxidative phosphorylation. Accordingly, the kidney contains one of the highest mitochondrial densities of any organ, and mitochondrial function remains essential for renal homeostasis under both physiological and diabetic conditions [5,6]

Even before the clinical onset of microalbuminuria, proximal tubular cells in both humans and animal models are exposed to profound metabolic stress caused by increased hyperabsorption, enhanced gluconeogenesis, and intrarenal hypoxia [7]. These disturbances impair mitochondrial oxidative phosphorylation, reduce ATP availability, and disrupt mitochondrial quality-control (MQC) mechanisms.

Damaged mitochondria, in turn, become major sources of reactive oxygen species (ROS), fueling oxidative stress, inflammation, fibrosis, and progressive nephron loss [8,9]. Mitochondrial fragmentation, impaired mitophagy, and reduced biogenesis are observed across different stages of DKD [10].

This emerging mitochondria-centric framework has broadened the understanding of DKD pathogenesis, aligning it with other microvascular complications of diabetes, where mitochondrial stress acts as a common pathogenic denominator. Given this central role, targeting mitochondrial dysfunction is now considered a promising strategy for early DKD intervention. However, the translation of mitochondrial biology into effective therapies remains incomplete and requires deeper mechanistic exploration in both preclinical studies and clinical settings [for recent review, see Ref. 11].

Despite advances in pharmacological management—including glycemic control, blood pressure reduction, and blockade of the renin–angiotensin system—current therapies slow but do not arrest the progression of DKD [12]. This therapeutic gap has stimulated interest in complementary strategies capable of targeting upstream metabolic and oxidative disturbances.

Among dietary interventions, Mediterranean diet (MD) has gained increasing attention and is recommended by the Kidney Disease Outcomes Quality Initiative as a preferred dietary pattern for individuals with CKD [13]. Characterized by high consumption of fruits, vegetables, legumes, whole grains, nuts, fish, and extra-virgin olive oil, and a low intake of red and processed meat, MD provides, among the others, a rich supply of polyphenols, monounsaturated and polyunsaturated fatty acids, and fiber. Epidemiological studies consistently associate adherence to MD with reduced incidence of CKD and slower decline in renal function [[14], [15], [16], [17]].

Emerging experimental evidence suggests that specific bioactive components abundant in the MD can modulate redox homeostasis, support mitochondrial quality in models of DKD [12]. These effects include enhancement of antioxidant defenses, reduction of mitochondrial ROS generation, and normalization of mitochondrial dynamics and biogenesis pathways. However, most mechanistic studies have evaluated isolated phytochemicals or extracts *in vitro* or in reductionist *in vivo* settings. Much less is known about whether a complex, whole-diet MD pattern can influence renal mitochondrial health or modify the course of DKD.

This narrative review aims to integrate existing clinical and experimental data to summarize how mitochondrial dysfunction contributes to DKD, focusing on early changes in podocytes and proximal tubular cells. We further discuss whether interventions such as adherence to the MD can beneficially modulate MQC mechanisms, potentially influencing disease onset and progression.

### 1.1 Methods

A structured, iterative literature search was conducted primarily in PubMed, focusing on four main domains: mitochondrial dysfunction and quality-control pathways in DKD; MD and kidney outcomes in humans; mitochondrial effects of MD-related bioactives; and the gut microbiota–metabolite–mitochondria axis in DKD. Search terms included combinations of “Mediterranean diet”, “diabetic kidney disease” and the earlier term “diabetic nephropathy”, “kidney function”, “mitochondria”, “mitochondrial dysfunction”, “oxidative stress”, “mitochondrial dynamics”, “mitophagy”, “mitochondrial biogenesis”, “polyphenols”, “gut microbiota”, and “microbial metabolites”. Human observational and interventional studies, together with relevant animal and renal cell studies, were considered, with particular attention to cell type-specific evidence distinguishing glomerular, particularly podocyte, from tubular, particularly proximal tubular cell, alterations whenever possible. The search primarily focused on studies published from 2021 onwards, while earlier mechanistically relevant or foundational studies were retained. Relevant reviews and their reference lists were also examined to identify pertinent primary studies. Articles were selected according to their clinical or mechanistic relevance to the predefined themes; studies unrelated to renal outcomes or the diet–mitochondria–DKD framework were excluded. Given the narrative design of the review, no formal systematic-review protocol was applied.

## 2 Mitochondrial dysfunction in early DKD

Renal cell types operate in distinct metabolic niches and differ markedly in mitochondrial content. Proximal tubular cells, among the most energy-dependent in the body, are mitochondria-rich, whereas podocytes and epithelial cells of the thin limb of Henle or collecting ducts contain far fewer. This heterogeneity underlies the compartment-specific vulnerability to early mitochondrial injury in DKD, including podocyte oxidative stress, tubular oxidative phosphorylation system (OXPHOS) deficits, and impaired glomerulo-tubular crosstalk [18,19].

### 2.1 Podocytes

Indirect evidence of mitochondrial dysfunction in podocytes in diabetic models has steadily accumulated [20,21]. Importantly, mitochondrial abnormalities have been directly documented in clinical samples from patients with DKD [6,22]. Several mitochondrial pathways have emerged as major contributors to podocyte injury, including excessive mitochondrial ROS production [23], disrupted mitochondrial dynamics [21], impaired mitochondrial biogenesis [5], and more recently, defects in mitochondrial DNA (mtDNA) replication and mitochondrial membrane remodeling [24,25].

#### 2.1.1 Oxidative stress

In podocytes, hyperglycemia induces excessive ROS production through over-reduction of the mitochondrial electron transport chain and NADPH oxidase activity, particularly NADPH oxidase 4 (Nox4) [26]. This is observed in cultured human and rodent podocytes and in diabetic mouse models, where elevated ROS cause mtDNA damage, lipid peroxidation, cytoskeletal alterations, and apoptosis. ROS also activate NLR family pyrin domain containing 3 (NLRP3) inflammasomes and NF-κB, amplifying inflammation and podocyte loss [27].

#### 2.1.2 Mitochondrial dynamics and mitophagy

Mitochondrial control of dynamics relays on the fission/fusion balance with fission being mediated by dynamin-related protein 1 (Drp1) and its receptors [mitochondrial fission 1 protein (Fis1), mitochondrial fission factor (Mff), and mitochondrial dynamics protein of 49 kDa and 51 kDa (MID49/51)], while fusion depending on optic atrophy 1 (OPA1), mitofusin 1 (Mfn1), and mitofusin 2 (Mfn2). Metabolic stress and ROS disrupt the physiological balance, with Drp1-mediated excessive fission contributing to mitochondrial fragmentation, reduced ATP production, and apoptosis in db/db and streptozotocin-diabetic mice [21]. Recent evidence shows that pathological cardiolipin remodeling mediated by acyl-CoA:lysocardiolipin acyltransferase-1 (ALCAT1) exacerbates mitochondrial injury. Increased oxidized cardiolipin and mitochondrial structural abnormalities were reported in DKD patients, db/db mice, and high glucose-treated podocytes, while ALCAT1 deficiency or cardiolipin-targeted therapy restored mitochondrial morphology and function [24]. Through AMP-activated protein kinase (AMPK)-dependent mechanisms, ALCAT1 also modulates regulators of mitochondrial dynamics, linking lipid remodeling to structural instability.

High glucose conditions impair PTEN-induced putative kinase 1 (PINK1)/Parkin-mediated mitophagy in podocytes, leading to accumulation of dysfunctional mitochondria [28]. Altered mitochondria associated membranes (MAM) structure further disrupts Ca²^+^ handling and autophagosome formation. ALCAT1-driven cardiolipin oxidation may also indirectly suppress mitophagy, given the requirement of cardiolipin for microtubule-associated proteins 1A/1B light chain 3B (LC3) recruitment to mitochondrial membranes [29,30].

#### 2.1.3 Mitochondrial biogenesis and protein quality control

Podocytes in diabetic models show reduced peroxisome proliferator-activated receptor gamma coactivator 1-alpha (PGC-1α) signaling and impaired mtDNA transcription and protein synthesis [31,32]. Recent studies have identified defective mtDNA replication as an additional contributor: A-kinase anchor protein 1 (AKAP1) overexpression in DKD enhances protein-chinasi C (PKC) -dependent phosphorylation of La-related protein 1 (Larp1), reducing mitochondria transcription factor A (TFAM) levels and thereby suppressing mtDNA replication and mitochondrial function [25]. AKAP1 knockdown or PKC inhibition rescues mtDNA replication and mitigates podocyte injury, positioning the AKAP1–PKC–TFAM axis as a novel target for preserving biogenesis.

Podocytes depend on efficient import and folding of nuclear-encoded mitochondrial proteins. High glucose impairs proteostasis, with selective heat shock protein (HSP) responses and activation of unfolded protein response- endoplasmic reticulum and UPR-mt under chronic stress, ultimately increasing vulnerability to apoptosis [33].

### 2.2 Tubular cells

Proximal tubular damage correlates strongly with renal functional decline and may even precede podocyte injury, as suggested by atubular glomeruli and by renal proximal tubular cells-derived nicotinamide mononucleotide inducing podocyte effacement [7,34]. Tubular atrophy and interstitial fibrosis further sustain inflammatory and profibrotic signaling [35]. Renal proximal tubular cells rely predominantly on mitochondrial OXPHOS (∼90% of ATP) and on the oxidation of fatty acids, lactate, and glutamine. In diabetes, increased substrate delivery, persistent ATP demand for transport, and enhanced gluconeogenesis create an imbalance between oxygen consumption and mitochondrial efficiency [36]. Hyperglycemia also perturbs glycolytic–mitochondrial crosstalk, further enhancing oxidative stress.

#### 2.2.1 Bioenergetics and oxidative stress

In renal proximal tubular cells, bioenergetic failure is now considered the upstream event that secondarily impairs mitochondrial dynamics, turnover, and renewal.

Diabetes reduces mitochondrial ATP efficiency and respiratory capacity, including decreased mitochondrial proteins and mtDNA content in urinary exosomes from DKD patients. Preclinical studies in insulin-resistant tissues and db/db mice further confirm reduced mitochondrial function, sometimes accompanied by compensatory increases in mtDNA driven by oxidative stress [37 and references within].

Hyperglycemia suppresses PGC-1α expression in Human Kidney-2 (HK-2) cells and streptozotocin-induced diabetic mice, leading to impaired fatty acid oxidation (FAO), increased ROS generation, mitochondrial fragmentation, apoptosis, and activation of profibrotic pathways such as transforming growth factor beta (TGF-β1). PGC-1α overexpression restores mitochondrial bioenergetics and attenuates tubular fibrosis [37]. Additional regulators modulate tubular metabolism under diabetic stress. Thiosulfate sulfurtransferase (TST) deficiency disrupts mitochondrial FAO and exacerbates tubular injury, whereas TST restoration or thiosulfate supplementation rescues mitochondrial function [38]. Mitochondrial oxidative stress also promotes lipid and sphingolipid accumulation in tubular cells, processes alleviated by the peptide Szeto-Schiller (SS)31 or Metrnl via sirtuin 3 (SIRT3)–AMPK/ uncoupling protein 1 (UCP1) signaling [39,40]. Hyperglycemia-induced ROS overproduction further suppresses antioxidant defenses, including manganese superoxide dismutase (MnSOD), while increasing cluster of differentiation 38 (CD38) expression, reducing the NAD^+^/NADH ratio, and impairing SIRT3 activity, thereby amplifying oxidative damage. Adaptive responses such as hypoxia-inducible transcription factor 1 (HIF-1α) activation can partially restore redox balance and mitigate tubular injury in diabetic models, highlighting the tight coupling between energy metabolism, oxidative stress, and tubular vulnerability in DKD [37 and references within].

#### 2.2.2 Mitochondrial dynamics and mitophagy

Hyperglycemia shifts the balance between fission and fusion toward fission, causing mitochondrial fragmentation and functional impairment in HK-2 cells. In DKD models, Drp1 is overactive and fusion proteins decline: AMPK–Phosphoglycerate Mutase Family Member 5 (PGAM5)–Drp1 signaling promotes injury; stromal cell-derived factor 1 (SDF-1α) /C-X-C chemokine receptor type 4 (CXCR4) inhibition enhances Drp1 and suppresses OPA1; high glucose exposure increases Drp1 and reduces Mfn2 in HK-2 cells [37]. Patient samples confirm increased tubular mitochondrial fragmentation. Runt-related transcription factor 3 (RUNX3) overexpression restores Mfn1/2 levels, reduces Drp1, and limits ROS and apoptosis, protecting tubular mitochondria [41]

Mitophagy is particularly active in renal proximal tubular cells. Hyperglycemia impairs mitophagy, contributing to mitochondrial fragmentation, defective turnover, and glucotoxic stress. Mechanisms include inositol oxygenase (MIOX) -mediated disruption of PINK1/Parkin-dependent mitophagy in HK-2 cells and streptozotocin mice, as well as impaired mitophagy via lncRNA nuclear enriched abundant transcript 1 (NEAT1), TIPE1, and mammalian target of rapamycin complex 1 (mTORC1) hyperactivation [37,42,43]. X-linked inhibitor of apoptosis (XIAP)- unc-51 like autophagy activating kinase 1 (ULK1) mediated mitophagy regulates carnitine metabolism, and its restoration mitigates tubular mitochondrial injury [43]. Overall, mitophagy interacts with mitochondrial dynamics and ROS regulation to preserve mitochondrial integrity under stress.

#### 2.2.3 Mitochondrial biogenesis

Altered mitochondrial biogenesis contributes significantly to tubular mitochondrial dysfunction in DKD. Hyperglycemia-induced downregulation of PGC-1α leads to reduced mitochondrial protein synthesis, impaired turnover, and disruption of mitochondrial homeostasis. Consistently, urinary exosome analyses from DKD patients reveal decreased markers of mitochondrial biogenesis, providing direct human evidence of impaired mitochondrial renewal. In contrast, compensatory increases in mtDNA have been observed in db/db mice, likely reflecting a stress-driven but functionally insufficient response [37 and references within]. Defective biogenesis exacerbates tubular susceptibility to metabolic stress and fibrosis. Genetic or functional disruption of key regulators, such as retinoic acid receptor α (RARα), further impairs mitochondrial function in proximal tubules, promoting apoptosis, tubular atrophy, and interstitial fibrosis in diabetic models [44]. Insufficient mitochondrial biogenesis, rather than simple mitochondrial loss, contributes to energy failure and progressive tubular injury in DKD.

Interestingly, accumulating evidence identifies mitochondrial dynamics, mitophagy, oxidative stress, energy metabolism, and stress-response pathways as convergent therapeutic targets across nephron compartments. However, translation to clinical practice remains limited, and future research should define optimal agents, precise molecular targets, timing, and predictive biomarkers for mitochondria-directed therapies in DKD [for recent review, see Ref. 11].

## 3 Linking MD and DKD: a strategy for prevention and slowing disease progression

Current DKD therapies rely on metabolic control and comorbidity management, with renin–angiotensin–aldosterone system blockers, sodium/glucose cotransporter 2 (SGLT2) inhibitors, non-steroidal mineralocorticoid receptor antagonists, and glucagon-like peptide-1 (GLP-1) receptor agonists showing renoprotective effects [12]. Nonetheless, tubular atrophy, interstitial fibrosis, and end-stage renal disease progression remain frequent, highlighting the need for novel interventions.

Lifestyle and dietary interventions play a central role in the prevention and clinical management of DKD, influencing both renal function decline and the onset of systemic complications. Nutritional therapy is increasingly recognized as an effective strategy to slow DKD progression, delay the transition to end-stage renal disease, and improve patients’ quality of life [45]. In this context, dietary patterns rich in plant-based foods, particularly MD, have attracted growing attention as potentially nephroprotective approaches.

Importantly, MD ingredients and food features align well with current dietary recommendations for patients with DKD. The relatively low intake of animal protein in MD results in total protein consumption close to the recommended ∼0.8 g/kg/day for DKD patients, while the emphasis on plant-based foods increases the intake of fibers, alkali, vitamins, and polyphenols [46]. These components contribute to improved metabolic control, attenuation of systemic inflammation, and maintenance of gut microbiota homeostasis, all of which are relevant to DKD pathophysiology. Based on this nutritional profile, the European Renal Association–European Dialysis and Transplant Association has endorsed the MD as the dietary pattern of choice for patients with CKD, including those with diabetes [18]. Concerns have historically been raised regarding the risk of hyperkalemia associated with increased consumption of fruits and vegetables in DKD. However, accumulating evidence indicates that plant-based potassium has lower bioavailability than potassium derived from food additives, and that higher vegetable intake is not necessarily associated with clinically relevant increases in serum potassium levels [47]. Moreover, the alkali load provided by plant foods may help counteract metabolic acidosis, a common complication in DKD [46]. Supporting these observations, a recent randomized crossover trial in patients with stage 3–4 CKD demonstrated that short-term adherence to a MD did not significantly alter serum or urinary potassium levels, confirming its safety in this population [48]. To further tailor this dietary pattern to renal patients, the concept of a Mediterranean Renal Diet has been proposed, combining MD principles with controlled protein, sodium, and phosphate intake [46].

### 3.1 Human studies

Available evidence from observational studies and randomized clinical trials supports an association between higher adherence to the MD and improved renal outcomes. A secondary analysis of the CORDIOPREV randomized controlled trial, including patients with coronary heart disease, showed that, over a 5-year follow-up, assignment to a MD was associated with a lower decline in estimated glomerular filtration rate (eGFR) and reduced urinary albumin-to-creatinine ratio (uACR), particularly in individuals with TD2 and obesity [15]. Similarly, a large prospective cohort study involving over 33,000 participants with hyperglycemia showed that higher adherence to an alternate MD score was associated with a significantly lower risk of developing DKD, with stronger protective effects observed in patients with established T2D [49].

Data from population-based cohorts further reinforce these findings. Analyses of National Health and Nutrition Examination Survey participants revealed that higher adherence to MD was associated with lower frailty scores and improved overall health status in individuals with CKD [50]. In line with this, a prospective analysis of the UK Biobank cohort, including more than 100,000 participants, demonstrated that greater adherence to a Mediterranean dietary pattern was associated with a reduced incidence of DKD over a median follow-up of more than nine years [16].

Collectively, these studies indicate that MD is associated with a lower risk of DKD onset and slower renal function decline (for a schematic summary, see Table 1). Proposed mechanisms underlying these benefits include improved glycemic control, reduced oxidative stress and systemic inflammation, favorable lipid profiles, lower blood pressure, and beneficial modulation of the gut microbiota [17,51]. However, available clinical evidence remains limited and is largely observational, and these studies do not provide direct insight into the subcellular or molecular mechanisms underlying the observed associations.

**Table 1:** Key recent human studies investigating the effects of MD on renal outcomes, metabolic parameters, and DKD-related complications.

Study	Design/Population	Intervention/Exposure	Duration	Main Outcomes	Key Findings	Reference
Podadera-Herreros et al., 2024	Prospective RCT, 1002 CHD patients with obesity/T2D	Mediterranean Diet vs. Low-Fat Diet	5 years	eGFR decline, uACR	MD reduced eGFR decline in T2D, lowered uACR in obese T2D	Podadera-Herreros et al., Nutr Diabetes. 2024;14:27 ^15^
Qu et al., 2024	Prospective cohort, 33,441 hyperglycemic participants	Adherence to MD (AMED score)	Median follow-up not specified	DKD incidence	Higher adherence to MD associated with lower DKD risk; effect stronger in T2D	Qu et al., BMC Med. 2024;22:224 ^49^
Peng et al., 2025	Observational National Health and Nutrition Examination Survey cohort, 4445 adults	MD Score, HEI-2020, other diet indices	2007–2016	Frailty scores, kidney health	Higher adherence to MD linked to lower frailty and better DKD health indicators	Peng et al., Front Nutr. 2025;12:1602587 ^50^
Maroto-Rodriguez et al., 2025	Prospective cohort, UK Biobank, 106,870 adults	aMED score	Median follow-up 9.27 years	DKD incidence	Highest adherence to aMED associated with lower risk of DKD (HR 0.84)	Maroto-Rodriguez et al., Am J Clin Nutr. 2025;121:445–453 ^16^
Chauveau et al., 2018	Review/Guidelines	MD for CKD/DKD	—	Multiple metabolic and renal outcomes	MD recommended for CKD/DKD; benefits include reduced inflammation, improved lipid profile, blood pressure, glucose control, and gut microbiota modulation	Chauveau et al., Nephrol Dial Transplant. 2018;33:725–735 ^17^
Cigarrán-Guldris et al., 2022	Review	Fiber intake within MD in CKD	—	Gut health, metabolic parameters	Fiber from plant-based MD foods supports gut microbiota, reduces inflammation	Cigarrán-Guldris et al., Nutrients. 2022;14:4419 ^51^

### 3.2 Preclinical studies

In contrast to the growing body of epidemiological and clinical evidence, experimental studies directly investigating MD as a whole in models of DKD are currently lacking. No animal or in vitro studies have specifically evaluated the renal effects of a complete MD dietary pattern, highlighting a critical gap between clinical observations and mechanistic research. Most preclinical evidence instead derives from studies investigating individual bioactive components characteristic of the MD.

In animal models of diabetes or kidney injury, olive oil–derived polyphenols have consistently demonstrated nephroprotective properties. Administration of triterpenoid-rich olive oil reduced oxidative stress, glomerular hypertrophy, and renal injury in diabetic mice [52], while hydroxytyrosol significantly attenuated proteinuria, glomerulosclerosis, and oxidative and nitrosative stress in streptozotocin diabetic rats [53]. Similar protective effects were observed with tyrosol, which improved renal morphology and antioxidant capacity in experimental diabetes [54].

Polyphenols, abundant in fruits and vegetables typical of MD, have also been extensively studied. A recent dose–response meta-analysis of animal models demonstrated that resveratrol, a stilbene abundantly found in grapes, significantly improves multiple indices of DN, including proteinuria, mesangial expansion, glomerular hypertrophy, and renal function parameters [55]. In diabetic mice, administration of the flavonol quercetin (abundant in onions and apples) ameliorated glomerular and tubular injury and limited extracellular matrix accumulation through modulation of inflammatory and apoptotic pathways [55,56].

Lipid components of the MD have similarly been explored in experimental settings. Omega-3 polyunsaturated fatty acids reduced renal fibrosis and inflammation in models of CKD by inhibiting macrophage activation and profibrotic signaling [57,58]. Lycopene, a carotenoid abundant in Mediterranean fruits and vegetables, attenuated diet-induced renal injury by suppressing inflammatory pathways in high-fat diet–fed mice [59].

In vitro studies further support these findings, showing that MD-derived bioactives can protect renal cells from hyperglycemia- or lipotoxicity-induced damage. Omega-3 polyunsaturated fatty acids enhanced antioxidant defenses in palmitate-treated podocytes via activation of nuclear factor-erythroid 2-related factor 2 (NRF2) signaling [60], while resveratrol, curcumin, and gallic acid prevented glyoxal-induced oxidative stress, mitochondrial dysfunction, and cell death in renal cells [61]. Additional studies demonstrated protective effects of eicosapentaenoic acid (EPA, a crucial omega-3 polyunsaturated fatty acids) and resolvin D1 on podocyte structure and inflammatory signaling under diabetic conditions [62,63].

Collectively, these experimental studies indicate that individual MD components exert consistent renoprotective effects in preclinical models. However, the absence of studies addressing MD as an integrated dietary pattern limits translational interpretation. This gap underscores the need to investigate how the combined dietary context of MD may converge on shared molecular targets—particularly mitochondrial pathways—relevant to DKD progression.

## 4 Mitochondria as targets of MD bioactives in DKD

While direct evidence linking the MD to mitochondrial protection in DKD remains limited, a growing body of literature highlights the potential of specific dietary polyphenols and bioactives in modulating MQC in renal cells. These compounds, abundant in MD foods such as, among the others, olive oil, legumes, and berries, exhibit multi-level protective effects spanning oxidative stress reduction, mitochondrial dynamics stabilization, biogenesis enhancement, and mitophagy activation, with effects observed in both glomerular and tubular compartments [61,64,65,[67], [68], [69], [70],^72^]. As discussed below, these direct cellular effects may coexist with systemic metabolic and microbiota-mediated mechanisms influencing renal mitochondrial homeostasis.

Resveratrol has been studied for its mitochondrial protective properties in DKD. In diabetic mice, resveratrol mitigates podocyte damage via SIRT1/PGC-1α–mediated attenuation of mitochondrial oxidative stress, restoring mitochondrial membrane potential and improving respiratory chain complex I and III activity [64]. Beyond glomerular effects, resveratrol preserves tubular epithelial cell viability by reducing apoptosis, ameliorating interstitial pathology, and preventing glycoxidative stress-induced mitochondrial dysfunction, as demonstrated in vitro in renal cell lines exposed to glyoxal [61]. Furthermore, resveratrol limits excessive mitochondrial fission via the phosphodiesterase-4 (PDE4D)/protein chinase A (PKA)/Drp1 axis, stabilizing glomerular mitochondrial networks and curbing propagation of mitochondrial injury under hyperglycemic conditions [65].

However, these experimental findings should be interpreted cautiously in the context of the MD. Grapes and wine represent major dietary sources of resveratrol, but the amounts provided by habitual dietary intake are substantially lower than the doses commonly used in experimental studies. Moreover, wine-derived resveratrol is consumed together with ethanol, whose established health risks preclude considering wine consumption as a strategy to reproduce the effects observed with isolated resveratrol [66]. Thus, these studies support the mechanistic potential of resveratrol rather than a specific contribution of wine to the renal benefits associated with the MD.

Myricetin, a flavonol present in berries and vegetables, activates mitophagy in podocytes through coordinated regulation of fosfoinositide 3-chinasi/ protein-chinasi B (PI3K/Akt) and PINK1/Parkin signaling pathways [67]. In high glucose/high lipid exposed MPC-5 cells, myricetin preserves mitochondrial morphology, reduces cristae disruption, and promotes autophagic clearance of dysfunctional mitochondria, indicating its capacity to counteract early podocyte mitochondrial stress in DKD [67].

Caffeic acid phenethyl ester, primarily acting on tubular epithelial cells, attenuates ferroptosis by restoring PINK1-mediated mitophagy, rescuing mitochondrial membrane potential, and reducing ROS accumulation under hyperglycemia [68].

Catechins, particularly (+)-catechin, reduce ER stress and NLRP3 inflammasome activation in proximal tubular cells, indirectly supporting mitochondrial homeostasis by decreasing ROS generation and preserving mitochondrial function [69].

Verbascoside, a caffeoyl phenylethanoid glycoside abundant in olive leaves, activates AMPK and inhibits NOX4/NF-κB signaling in proximal tubular cells, mitigating oxidative stress and fibrosis, and indirectly supporting mitochondrial integrity while potentially reducing tubular glucose overload through SGLT2 inhibition [70].

Finally, genistein, an isoflavone particularly abundant in soy but also occurring at lower levels in foods consumed within Mediterranean dietary patterns [71], improves mitochondrial function in diabetic rats by modulating mitogen-activated protein kinases (MAPK) /NF-κB signaling and enhancing Mfn2 expression, stabilizing mitochondrial dynamics, reducing ROS, and preserving podocyte and tubular structure [72].

Collectively, these polyphenols converge on mitochondrial mechanisms including oxidative stress attenuation, promotion of mitophagy, stabilization of dynamics, and enhancement of biogenesis, with complementary effects in glomerular and tubular compartments (for a schematic summary, see Table 2).

**Table 2:** MD–derived bioactive compounds and their reported effects on glomerular and tubular mitochondrial pathways in experimental models of DKD.

Bioactive compound	Main dietary source (MD)	Experimental model	Podocyte effects	Tubular effects	Mechanisms	Reference
Resveratrol	Grapes	Diabetic mice; renal cell lines	↓ROS, ↑biogenesis, ↑OXPHOS, ↓fission	↓ROS, ↓apoptosis, ↑ATP	SIRT1/PGC-1α, PDE4D/PKA, Drp1	Zhang et al., 2019 ^64^; Hashemzaei et al., 2020 ^61^; Zhu et al., 2023 ^65^
Myricetin	Berries, vegetables	MPC-5 podocytes (high glucose / high lipid)	↑mitophagy, ↑autophagy flux, ↓cristae disruption	–	PI3K/Akt, PINK1/Parkin	Liu et al., 2026 ^67^
Caffeic acid phenethyl ester (CAPE)	Propolis-derived polyphenol	Tubular epithelial cells (high glucose)	–	↓ROS, ↑mitophagy, ↓ferroptosis	PINK1	Lu et al., 2025 ^68^
Catechins	Tea, fruits	Diabetic mice; HK-2 cells	–	↓ROS, ↓ER stress	NLRP3, indirect mito protection	Zhang et al., 2024 ^69^
Verbascoside	Olive leaves, plant foods	Proximal tubular cells	–	↓ROS, ↑AMPK activation	NOX4/NF-κB	Ahmed et al., 2025 ^70^
Genistein	Legumes (e.g., chickpeas, beans, lentils; low amounts)	Diabetic rats	↓ROS, ↑Mfn2, ↓apoptosis	↓ROS, ↑Mfn2	MAPK/NF-κB	Li et al., 2022 ^72^

Beyond direct cellular effects, systemic mediators—including gut microbiota-derived metabolites and circulating metabolic regulators—play a crucial role in maintaining mitochondrial integrity in DKD. Gut microbial metabolites, such as indole-3-propionic acid (IPA), exemplify this axis: IPA protects glomerular endothelial mitochondria by preventing SIRT1 ubiquitination and proteasomal degradation. In diabetic mice, IPA supplementation restores SIRT1-mediated PGC-1α activation, enhancing mitochondrial biogenesis and antioxidant defenses, which translates into improved glomerular filtration and reduced albuminuria [73,74]. These findings underscore a functional gut microbiota–mitochondria axis, in which microbial metabolites directly influence MQC in the kidney.

Systemic metabolic mediators further shape tubular mitochondrial health. D-amino acids, recently recognized as biologically active circulating metabolites, modulate mitochondrial function by regulating ROS production, oxidative stress responses, and cellular proliferation, thereby influencing tubular integrity and resilience [75]. Similarly, branched-chain amino acid catabolism exerts a significant impact on tubular mitochondria: impaired branched-chain amino acid breakdown compromises mitochondrial density and function, whereas inhibition of branched-chain α–ketoacid dehydrogenase complex kinase restores mitochondrial activity and, when combined with SGLT2 inhibition, reduces proteinuria and kidney hypertrophy [76].

Mitochondrial fatty acid metabolism also contributes to systemic regulation of tubular resilience. Deficiency of TST, a key mitochondrial enzyme for fatty acid oxidation, exacerbates tubular injury and fibrosis by impairing mitochondrial FAO. Restoration of TST expression or sodium thiosulfate supplementation rescues FAO and improves tubular mitochondrial function, highlighting the importance of metabolic flexibility in mitigating DKD progression [38].

Collectively, these observations reveal a multilayered systemic influence on renal mitochondria, encompassing gut microbiota-derived metabolites, circulating amino acids, and metabolic enzymes. Integration of these pathways suggests that the protective effects of MD bioactives may extend beyond direct mitochondrial modulation to include enhancement of systemic metabolic signals and microbial metabolite production. This perspective reinforces the concept of MD as a “mitochondria-directed” nutritional strategy, capable of preserving renal mitochondrial health across both glomerular and tubular compartments.

## 5 Limitations of current evidence

Despite increasing interest in nutritional modulation of MQC in DKD, the evidence remains limited and fragmented. A major gap is the absence of studies assessing MD as an integrated dietary pattern with mitochondrial endpoints. Thus, the molecular impact of long-term MD adherence on glomerular and tubular mitochondrial biology is essentially unknown. Current mechanistic insights rely almost exclusively on isolated polyphenols administered at pharmacological concentrations, which do not recapitulate the complexity, food matrix interactions, or physiological exposure typical of MD consumption. Moreover, major MD components—such as unsaturated fatty acids, dietary fiber, olive oil bioactives, and microbiota-derived metabolites—are largely unexplored in the context of renal mitochondrial function in DKD. Finally, available data rely predominantly on experimental models with heterogeneous mitochondrial readouts, while clinical studies rarely incorporate mitochondrial biomarkers, limiting translational relevance. Together, these gaps highlight the need for integrated, diet-centered approaches to clarify whether MD can be considered a true mitochondria-targeting strategy in DKD.

## 6 Conclusions and perspectives

**Fig. 1:**
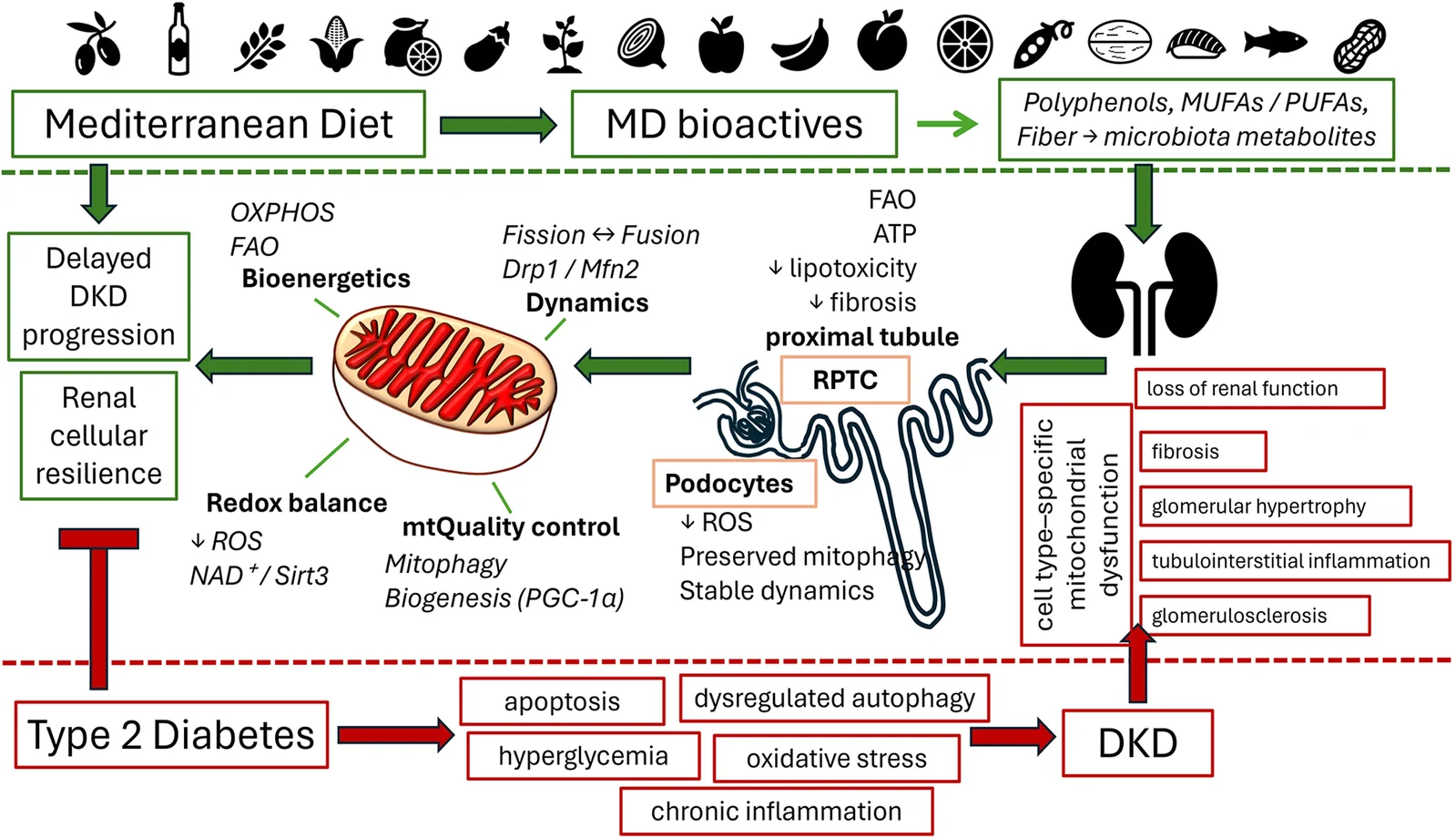
The Mediterranean diet–mitochondria axis in DKD. Hyperglycemia induces early, cell type–specific mitochondrial dysfunction, with podocytes developing excessive mitochondrial ROS, altered dynamics, and defective mitophagy, and proximal tubular cells exhibiting bioenergetic failure, impaired fatty acid oxidation (FAO), and disrupted mitochondrial (mt) quality control. Clinical and epidemiological studies associate higher adherence to Mediterranean diet (MD) with preserved renal function and reduced risk of DKD progression. Mechanistically, MD–derived bioactives may act at both systemic and renal levels to counter these compartment-specific mitochondrial alterations, promoting redox balance, mitochondrial homeostasis and cellular resilience.

Mounting evidence supports mitochondrial dysfunction as a unifying mechanism in the initiation and progression of DKD, affecting both glomerular and tubular compartments. Within this framework, MD emerges not merely as a cardiometabolic-friendly dietary pattern, but as a potential modulator of renal cellular resilience. Available human studies suggest an association between higher MD adherence and favorable renal outcomes, while experimental evidence indicates that selected MD-related bioactives can modulate mitochondrial pathways, including redox homeostasis, dynamics, mitophagy, and biogenesis. (for a schematic representation, see Fig. 1).

Importantly, these effects are unlikely to be driven by single nutrients in isolation. Rather, MD should be viewed as a complex nutritional ecosystem in which polyphenols, unsaturated fatty acids, fiber, and microbiota-derived metabolites act synergistically to preserve mitochondrial integrity under diabetic stress.

Future research should move beyond reductionist approaches and investigate whole-diet interventions incorporating clinically accessible mitochondria-related biomarkers alongside omics-based profiling, and gut microbiota signatures. Potential candidates include mitochondrial membrane potential and mass in peripheral blood cells [77] and plasma acylcarnitine profiles reflecting mitochondrial fatty acid metabolism [78]. Whether these markers are responsive to dietary interventions and accurately reflect renal mitochondrial dysfunction remains to be established. Longitudinal studies should determine whether changes in these markers track MD adherence and precede or parallel conventional renal outcomes. Targeting the MD–mitochondria axis may therefore represent a systems-level strategy to complement pharmacological therapies and support renal resilience in DKD.

## CRediT authorship contribution statement

Giovanna Mercurio: Conceptualization, Investigation, Writing – original draft, Writing – review & editing; Antonia Giacco: Writing – review & editing; Nicla Scopigno: Writing – review & editing; Michela Vigliotti: Writing – review & editing; Giuseppe Petito: Writing – review & editing; Maria Moreno: Writing – review & editing; Federica Cioffi: Writing – review & editing; Elena Silvestri: Conceptualization, Writing – original draft, Writing – review & editing, Supervision.

## Informed consent

Not applicable.

## Studies on human and/or animal statement

This article is a narrative review and does not report original research involving human participants or animals.

## Ethical approval and registration number

Not applicable.

## Declaration of Generative AI and AI-assisted technologies in the writing process

The authors have not used any AI.

## Funding

This research did not receive any specific grant from funding agencies in the public, commercial, or not-for-profit sectors.

## Data availability statement

No new data were generated or analyzed in this study. Data sharing is therefore not applicable to this article.

## Declaration of competing interest

The authors declare that they have no known competing financial interests or personal relationships that could have appeared to influence the work reported in this paper.

## Glossary

AKAP1: A-kinase anchor protein 1
ALCAT1: Acyl-CoA:lysocardiolipin acyltransferase-1
AMPK: AMP-activated protein kinase
CD38: cluster of differentiation 38
CXCR4: C-X-C chemokine receptor type 4
DKD: Diabetic Kidney Disease
DRP1: dynamin-related protein 1
eGFR: estimated glomerular filtration rate
EPA: eicosapentaenoic acid
FAO: fatty acid oxidation
Fis1: mitochondrial fission 1 protein
GLP-1: glucagon-like peptide-1
HIF-1α: hypoxia-inducible transcription factor 1
HK-2: Human Kidney-2
HSP: heat shock protein
IPA: indole-3-propionic acid
Larp1: La-related protein 1
LC3: microtubule-associated proteins 1A/1B light chain 3B
MAM: mitochondria associated membranes
MAPK: mitogen-activated protein kinases
MD: mediterranean diet
Mff: mitochondrial fission factor
MID49/51: mitochondrial dynamics protein of 49 kDa and 51 kDa
MIOX: inositol oxygenase
MQC: mitochondrial quality control
mtDNA: mitochondrial DNA
mTORC1: mammalian target of rapamycin complex 1
NEAT1: nuclear enriched abundant transcript 1
NLRP3: NLR family pyrin domain containing 3
Nox4: NADPH oxidase 4
NRF2: Nuclear factor-erythroid 2-related factor 2
OXPHOS: oxidative phosphorylation system
PDE4D: phosphodiesterase-4
PGAM5: Phosphoglycerate Mutase Family Member 5
PGC1α: peroxisome proliferator-activated receptor gamma coactivator 1-alpha
PI3K/Akt: fosfoinositide 3-chinasi/ protein-chinasi B
PINK1: PTEN-induced putative kinase 1
PKA: protein chinase A
PKC: protein-chinasi C
RARα: retinoic acid receptor α
ROS: reactive oxygen species
RUNX3: runt-related transcription factor 3
SDF-1α: stromal cell-derived factor 1
SGLT2: sodium/glucose cotransporter
SIRT1/3: sirtuin 1/3
SS31: (peptide) Szeto-Schiller 31
T2D: type 2 diabetes
Tfam: mitochondria transcription factor A
TGF-β1: transforming growth factor beta
TST: thiosulfate sulfurtransferase
uACR: urinary albumin-to-creatinine ratio
UCP1: uncoupling protein 1
ULK1: unc-51 like autophagy activating kinase 1
XIAP: X-linked inhibitor of apoptosis
